# ERβ as a mediator of estrogen signaling in inflammatory breast cancer

**DOI:** 10.18632/oncotarget.28425

**Published:** 2023-06-12

**Authors:** Harika Nagandla, Christoforos Thomas

**Keywords:** inflammatory breast cancer, IBC, ERβ, metastasis, actin based cell migration

Inflammatory breast cancer (IBC) is a rare and aggressive form of breast cancer which accounts for 2–4% of all new breast cancer cases detected in the United States [1]. Even with the application of standard multi-modality treatment approach that incorporates neo-adjuvant chemotherapy, radiation and surgery, the 5-year survival rate for IBC is only about 40–50% [2]. Breast cancer can be typically stratified into different types based on the presence of molecular drivers such estrogen receptor (ERα), progesterone receptor (PR) or human epidermal growth factor receptor 2 (HER2), which inform the treatment choice. For IBC, there is a substantially higher incidence of ERα negativity compared with other forms of breast cancer that can reach up to 60% [3] and a specific targetable driver signaling pathway has not been identified so far. About one in three patients already have distant metastasis at the time of diagnosis, contributing to the aggressiveness and poor outcomes associated with IBC [2].

Despite the absence of ERα from the majority of IBC tumors estrogen signaling has been implicated in progression of the disease through ERα-independent pathways [4]. ERβ is a ligand activated transcription factor that mediates effects of estrogen, along with ERα in different tissues during growth and development by regulating transcription of target genes. Tumor suppressive effects of ERβ have been documented in diverse cancer types such as thyroid, kidney, prostate, glioblastoma, ovarian and breast cancer [5, 6]. A previous scientific report from our group was the first to look at ERβ levels in clinical specimens of IBC patients [6]. Immunohistochemical analysis of IBC tumors revealed the association of higher expression of ERβ with significant improvement in metastasis free survival. Reinforcing this finding, analysis of an IBC patient dataset also showed strong association between high tumor mRNA levels of ERβ and better overall survival. These clinical associations reflected the potential of tumor ERβ to serve as a biomarker for better prognosis in IBC. They also triggered a series of preclinical studies to test whether ERβ and its agonists inhibit metastasis of IBC tumors.

An initial assessment of the morphology and protein expression of ERβ in several IBC cell lines including FC-IBCO2, KPL4, SUM149, IBC-3, SUM-190 and BCX-010 indicated a correlation between lower protein levels of ERβ and increased migratory phenotype of the cells in culture. For further *in vitro* analysis, two clones of KPL4 cells with ERβ knockout were generated and in one of these clones the receptor was re-expressed to serve as an additional control. As expected, the ERβ knockout cells exhibited significantly higher rates of migration and invasion compared to the cells with the endogenous receptor and the knockout cells with re-introduced ERβ. Similar to upregulation, treatment of ERβ proficient cells with the ERβ specific agonist LY500307 led to reduced invasion in a dose dependent manner, while showing no effect in ERβ knockout KPL4 cells. Similarly, FC-IBCO2 cells with depleted ERβ also exhibited increased invasiveness compared to the control cells.

Orthotopically and intravenously injected ERβ knockout KPL4 cells also exhibited higher rates of metastasis in lungs of immune deficient mice compared with the ERβ proficient cells, as observed through *in-vivo* bioluminescence imaging and histological examination of the resected lungs. In addition tο lungs, immunofluorescence analysis of bone marrow detected more tumor cells in bones of mice that were orthotopically implanted with ERβ knockout cells. The therapeutic relevance of these findings was investigated with the ERβ agonist LY500307. *Ex-vivo* bioluminescence imaging of lungs dissected out of vehicle- and LY500307-treated ovariectomized mice bearing orthotopic IBC tumors showed significantly less lung metastasis in ERβ agonist-treated mice.

The mechanism of the anti-metastatic activity of ERβ was investigated using high throughput gene expression and functional analysis of IBC cells with different ERβ levels. Genes associated with actin-based cell migration were found to be enriched in ERβ knockout KPL4 cells through micro-array analysis. The occurrence of actin cytoskeleton reorganization in absence of ERβ was further corroborated by the increased immunofluorescent staining for polymerized actin and the focal adhesion inducer vinculin in ERβ knockout cells that are both required for cytoskeleton remodeling in motile cells. Consistent with the formation of actin stress fibers, a Rho GTPase assay showed substantial increase in active (GTP bound) form of the cytoskeleton remodeler RhoC upon ERβ knockout, and a significant decrease following treatment of ERβ proficient cells with the agonist LY500307. Because RhoC was previously implicated in migration of IBC cells [7, 8] and its depletion reversed the increased invasion of ERβ knockout IBC cells it was suggested as an essential driver of the migratory phenotype of IBC cells in absence of ERβ.

**Figure 1:**
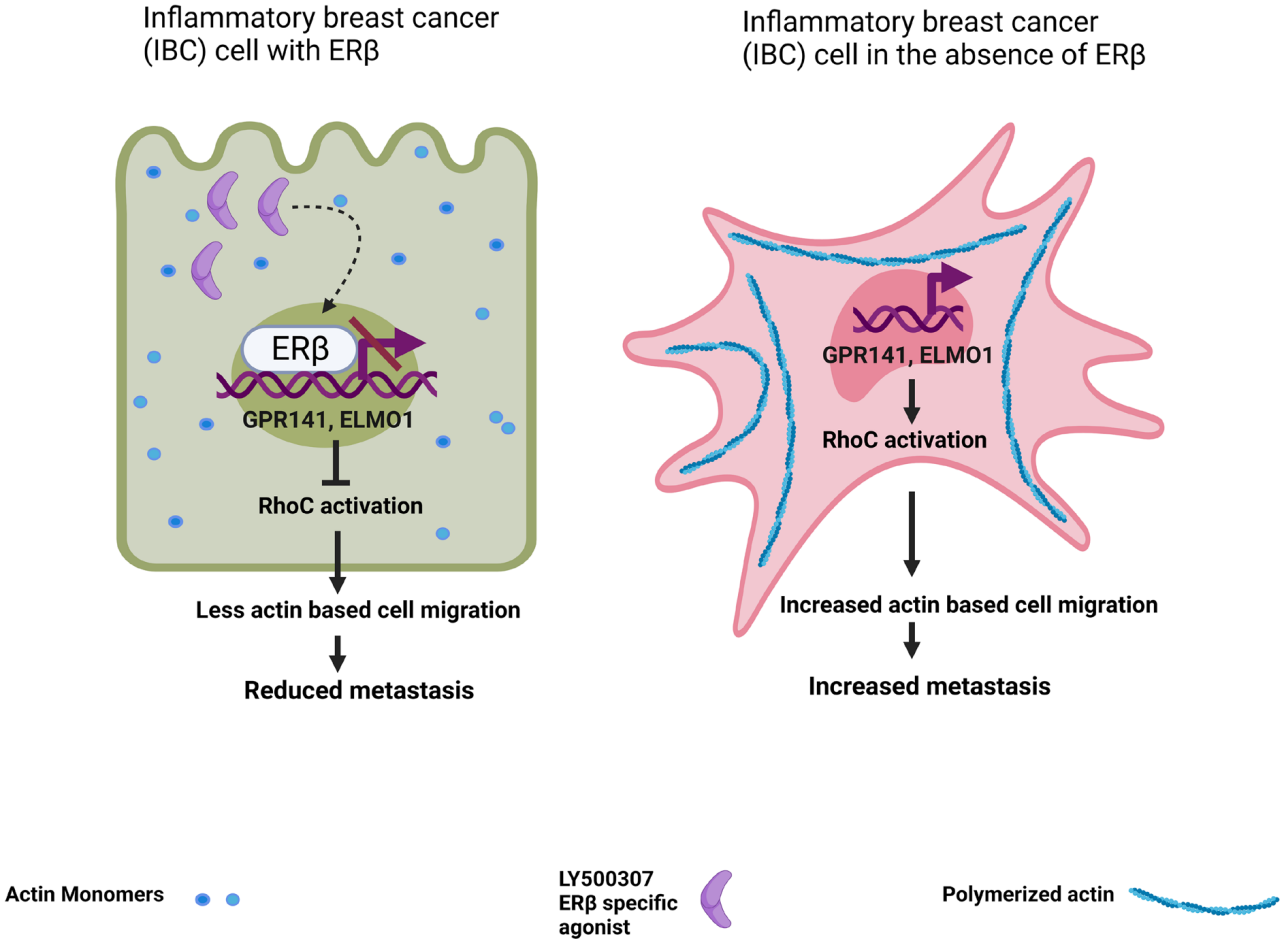
Upon activation by the specific agonist LY500307 ERβ binds to estrogen receptor binding elements (ERE) in regulatory regions of GPR141 and ELMO1 genes and suppresses their transcription, which in turn leads to diminished RhoC activation, reduced actin-based cell migration and metastasis. On the other hand, absence of ERβ in IBC cells leads to higher GPR141 and ELMO1 transcript and protein levels resulting in higher RhoC activation causing enhanced actin polymerization and migration which ultimately leads to increased metastasis.

G protein coupled receptors (GPCRs) are known to activate RhoGTPases through GEFs (Guanine nucleotide exchange factors) which catalyze exchange of GDP (inactive form) to GTP (active form) [9]. Turning to this pathway to identify ERβ-associated regulators of RhoC, authors focused on the GEF interacting protein ELMO1 that is required for the function of GEFs and the GPCR GPR141. As these genes were found in microarray analysis to be upregulated in ERβ knockout IBC cells, they were considered potential repressed targets of ERβ that are overexpressed in absence of the receptor to activate RhoC and increase the motility of IBC cells (Figure 1). The relevance of these genes for the biology of IBC metastasis was manifested by two major observations. First, their mRNA and protein levels were selectively upregulated in IBC cell lines. Second, their expression was found to be inversely proportional to ERβ in IBC cell models and their knockdown significantly reduced the invasive potential of ERβ knockout cells. Further, the protein levels of active RhoC, ELMO1 and phosphorylated Akt were greatly diminished in ERβ knockout IBC cells upon depletion of GPR141 clearly establishing the function of ELMO1 and RhoC downstream of GPR141 in the pathway that regulates migration in IBC cells.

Genomic analysis revealed the presence of estrogen response elements (EREs) in the regulatory regions of GPR141 and ELMO1 genes that are located next to each other on chromosome 7 in opposite orientation. Strong binding of ERβ to the promoters of GPR141 and ELMO1 was detected through ChIP-qPCR and this association was further enhanced in the presence of increasing concentrations of the ERβ agonists LY500307 and estrogen, demonstrating the direct regulation of GPR141 and ELMO1 by ERβ in IBC cells (Figure 1).

The work from our group [6] establishes ERβ as a tumor suppressor in IBC by demonstrating its strong antimetastatic activity in preclinical models of the disease and delineating the mechanism of action. The findings of our study also shed new light onto the biology of IBC metastasis by discovering new roles for estrogen signaling in disease progression and identifying novel responsive genes that may function as drivers for aggressive phenotypes. The discovery of new associated factors prompts additional research to evaluate their power as complementary biomarkers in prognosis and molecular targets that may lead to new treatments to overcome resistance and prolong survival in patients. But most of all, this work represents a timely manifestation of the major function of ERβ in cancer. The study provides abundant evidence to validate the previously reported anti-metastatic activity in breast cancer by employing models of aggressive disease and connecting this function with a more physiological role of the receptor in differentiation through the regulation of a complex network of developmental genes. The translational relevance of our work is underscored by the observed clinical associations that link ERβ to better clinical outcome in patients with IBC. These correlations warrant further investigation to evaluate the use of ERβ as a potential viable marker to stratify patients that are not likely to respond to standard therapy and require additional treatments. They have also set the stage for exploration and testing of highly specific agonists of ERβ in the clinic to curb high rates of metastasis observed in inflammatory breast cancer. Considering that a significant percentage of these cancers belong to clinical HER2-positive and TNBC phenotypes these ligands have the potential to advance treatments that can benefit patients with breast cancer of either subtype.
